# The widespread dissemination of integrons throughout bacterial communities in a riverine system

**DOI:** 10.1038/s41396-017-0030-8

**Published:** 2018-01-26

**Authors:** Gregory C. A. Amos, Semina Ploumakis, Lihong Zhang, Peter M. Hawkey, William H. Gaze, Elizabeth M. H. Wellington

**Affiliations:** 1 School of Life Sciences, University of Warwick, Coventry, UK; 2 European Centre for Environment and Human Health, University of Exeter Medical School, Knowledge Spa, Royal Cornwall Hospital, UK; 3 University of Birmingham, Birmingham, UK

## Abstract

Anthropogenic inputs increase levels of antimicrobial resistance (AMR) in the environment, however, it is unknown how these inputs create this observed increase, and if anthropogenic sources impact AMR in environmental bacteria. The aim of this study was to characterise the role of waste water treatment plants (WWTPs) in the dissemination of class 1 integrons (CL1s) in the riverine environment. Using sample sites from upstream and downstream of a WWTP, we demonstrate through isolation and culture-independent analysis that WWTP effluent significantly increases both CL1 abundance and antibiotic resistance in the riverine environment. Characterisation of CL1-bearing isolates revealed that CL1s were distributed across a diverse range of bacteria, with identical complex genetic resistance determinants isolated from both human-associated and common environmental bacteria across connected sites. Over half of sequenced CL1s lacked the 3′-conserved sequence ('atypical’ CL1s); surprisingly, bacteria carrying atypical CL1s were on average resistant to more antibiotics than bacteria carrying 3′-CS CL1s. Quaternary ammonium compound (QAC) resistance genes were observed across 75% of sequenced CL1 gene cassette arrays. Chemical data analysis indicated high levels of boron (a detergent marker) downstream of the WWTP. Subsequent phenotypic screening of CL1-bearing isolates demonstrated that ~90% were resistant to QAC detergents, with *in vitro* experiments demonstrating that QACs could solely select for the transfer of clinical antibiotic resistance genes to a naive *Escherichia coli* recipient. In conclusion, this study highlights the significant impact of WWTPs on environmental AMR, and demonstrates the widespread carriage of clinically important resistance determinants by environmentally associated bacteria.

## Introduction

Antimicrobial resistance (AMR) is a worldwide health issue, with forecasts of prevalent untreatable infections within the next decade [1]. Widespread AMR in the clinic is primarily a result of horizontal gene transfer which allows the mobilisation of resistance genes between pathogens and other bacteria [2]. Perhaps the best characterised genetic element associated with AMR is the class 1 integron (CL1), which has been proven to be a proxy for total AMR load [3]. CL1s are vehicles for adaptive genes, with the ability to capture and integrate mobile gene cassettes into a variable region where they are expressed under a common promoter [4]. Gene cassettes can confer several phenotypes including resistance to a broad range of antibiotic classes and the ability to survive exposure to biocides such as quaternary ammonium compounds (QACs) [5].

The ability to acquire multi-resistant phenotypes makes CL1s important genetic elements in the dissemination of AMR, however, it is likely that to date, their description and understanding in natural populations have not been fully explored. CL1s can contain a 3′-conserved segment (CS) region which has a partially deleted but functional QAC efflux pump *(qacE∆1*) fused to a sulphonamide resistance gene (*sul1*) [6]. The terminology 3′ ‘Conserved Segment’ is misleading as this sequence is not conserved throughout all CL1s. Most techniques analysing CL1 gene cassette diversity focus on amplification through the *intI1* to 3′-CS region, thus, atypical CL1s (CL1s without a 3′-CS region) and associated gene cassettes are missed [7–9]. Although limited studies have proved to be successful in amplifying the atypical CL1 variable region from human and animal isolates [9–11], to date, there is no established method for analysing atypical CL1s, nor any studies on their distribution, prevalence and contribution to AMR in the wider environment.

Environmental bacteria are increasingly recognised as playing a role in the development of resistance in the clinic via mobilisation of novel resistance genes such as the well-characterised examples of *qnr* and *bla*_CTX-M_ [12, 13]. In addition, environmental reservoirs of AMR can pose a risk to human health through potential exposure events, such as during recreational activities in polluted aquatic environments [14, 15]. Anthropogenic inputs to the environment are hypothesised to be the drivers of environmental AMR, with agricultural run-off and waste water treatment plant effluent (WWTP), both proven to increase AMR load [16–19]. However, the selective agents driving environmental AMR are not well characterised. Due to the many resistance genes CL1s contain, their persistence may be influenced by antibiotic residues, metals or biocides with selection of one cassette gene ‘co-selecting’ for others [20, 21]. Despite the importance of environmental bacteria in the dissemination of AMR, to date, we still do not fully understand the extent of the spread of resistance genes in environmentally associated bacteria, how this is impacted by anthropogenic inputs or the extent to which human-associated and common environmental isolates share resistance genes [22].

Our previous work has demonstrated WWTPs to be the key predictor variable for estimating AMR levels in aquatic systems [3]. The aim of the current study was to characterise the role of WWTP effluent in the formation and persistence of reservoirs of CL1s in the wider environment, and to broadly understand the distribution of different CL1 subsets and their contributions to resistance load in the riverine environment. Through the isolation of a wide range of Gram-negative bacteria with concurrent total community DNA analyses, this study provides a comprehensive investigation of the abundance and diversity of the unbiased CL1 community in both WWTP-impacted and unimpacted sediments. Results  presented here demonstrate the impact of WWTP effluent on CL1 and AMR gene dissemination throughout environmental bacteria (bacteria commonly found in the environment, not associated with the clinic), highlight a previously unrecognised source of resistance genes in the form of atypical CL1s and determine the role of QAC detergents in the selection and transfer of CL1s and AMR genes.

## Methods

### Sampling

Sampling took place in January 2011. Triplicate sediment core samples were taken from the River Sowe in the West Midlands, UK, at six different sites. The sites were 300 m, 600 m and 900 m upstream (US) and, 300 m, 600 m and 900 m downstream (DS) of a large urban (450,000 population estimate) tertiary WWTP as previously described [16, 19]. Sediment core samples were taken using a custom-made corer, allowing the top ~5 cm of sediment to be collected [3, 16, 19]. Upstream of the WWTP, geospatial mapping has previously shown that there were no WWTP inputs for >10 km, with Arable and Horticulture Grassland, and Improved Grassland surrounding the upstream  river stretch [3]. Sediment samples were stored at 4 °C post sampling and processed within 24 h of samples being collected [3].

### Total community CL1 analysis

Total community (metagenomic) DNA was extracted from the triplicate sediment samples taken at the six sample sites DS3 (furthest downstream of WWTP Finham), DS2, DS1, US1, US2 and US3 (furthest upstream of WWTP Finham). DNA was extracted using FASTDNA Spin kit for soil (MP Biomedicals) as per the manufacturer’s instructions, as previously described for river sediment [3]. For enumeration of total bacterial load, CL1s and resistance genes at each sediment site, quantitative PCR (qPCR) was performed using primers targeting the CL1 integrase, 16S rRNA and QAC resistance genes as published (Supplementary Information) [23]. Molecular prevalence was calculated by dividing the number of target genes by the number of 16 S rRNA copies, with corrections made for 16S rRNA copy number (mean 2.5 per genome, as previously described [23]).

### Bacterial enumeration and isolation

For cultivation of Gram-negative isolates, sediment from DS sites was pooled in equal parts (1 g total) and resuspended in 1 mL of PBS buffer, as previously described [16]. This was repeated for US samples. Chromocult Coliform Agar (Merck Millipore) was prepared as per the manufacturer’s instructions and amended with streptomycin (16 mg L^−1^), gentamicin (4 mg L^−1^), chloramphenicol (16 mg L^−1^), trimethoprim (4 mg L^−1^), sulphachloropyridazine (8 mg L^−1^), cefotaxime (2 mg L^−1^) or ceftazidime (16 mg L^−1^) with levels of antibiotics chosen in accordance with the standards set out by British Society for Antimicrobial Chemotherapy (BSAC) and Clinical and Laboratory Standards Institute (CLSI) [24]. DS and US samples were plated (200 µL) in triplicate for each antibiotic on unamended Chromocult and then incubated for 24 h at 30 °C. Viable plate counts were taken; blue colonies indicated presumptive *Escherichia coli*, pink colonies indicated all other coliforms which were not *E*. *coli* (presumptive coliforms excluding *E. coli*, PCEs) and white or other coloured colonies indicated other Gram-negative bacteria which were not coliforms (non-coliforms). Controls for performance of Chromocult agar at 30 °C were performed as previously described [16]. Resistant quotients (RQs) were calculated using Eq. (1).

$${\mathrm {Resistant}}\,{\mathrm{quotient}}=\frac{No.\,of \, resistant \, bacteria}{Total \, no. \, of \, bacteria}{\times} 100$$

Non-coliforms, PCEs and *E. coli* isolates were selected and purified up to a total of ten colonies for each bacterial group per selective plate, as previously described [16]. In cases where numbers were low, the maximum number of available colonies was selected. For unammended Chromocult plates; 50 non-coliforms, 50 PCEs and 10 *E. coli* isolates were selected and purified.

### Class 1 integron characterisation and isolate identification

All isolates were screened for the presence of the CL1 integrase gene (*intI1*) using primers IntA 5′-ATCATCGTCGTAGAGACGTCGG-3′ and IntB 5′-GTCAAGGTTCTGGACCAGTTGC-3′ as published [7]. CL1-containing isolates were initially characterised using published primers HS915 5′-GTGCCGTGATCGAAATCCAG-3′ and HS550 5′-CTAGGCATGATCTAACCCTCGG-3′ [25], with conditions optimised for long-range PCR (Supplementary Information). PCR products were Sanger sequenced by Macrogen (Korea). Analysis of non-clinical CL1s was performed using a modified integron-specific long-range two-step gene-walking method [26] (Supplementary Information) and sequencing (Macrogen, Korea). Bacteria were identified by sequencing PCR products obtained using the universal 16S rRNA primers 27 F 5′-AGAGTTTGATCMTGGCTCAG-3′ and 1525 R 5′-AGGAGGTGATCCAGCC-3′, with further identification of the Enterobacteriaceae by partial sequencing of dnaJ using primer pair DN1-1F 5′-ATYTRCGHTAYAACATGGA-3′ and DN1-2R 5′-TCACRCCRTYDAAGAARC-3′ as previously described [15, 27]. *Aeromonas* spp. were identified using partial sequencing of *gyrB* using primer pair gyrB3F 5′-TCCGGCGGTCTGCACGGCGT-3′ and gyrB14R 5′-TTGTCCGGGTTGTACTCGT-3′ [28]. *E*. *coli* was sequence typed using the Achtman multilocus sequence typing (MLST) scheme [29].

### Antimicrobial susceptibility testing

All CL1-positive isolates were tested for their susceptibility to the antibiotics ciprofloxacin, oxacillin, sulfamethoxazole, ertapenem, tetracycline, co-amoxiclav, streptomycin, cefpodoxime, cefuroxime, gentamicin, trimethoprim and tigecycline. Susceptibility was tested using the BSAC standardised disc susceptibility method as published [24].

### QAC resistance screening and transfer experiments

Characterising resistance to QAC detergents was performed as previously described [20] on a panel of CL1-positive isolates resistant to third generation cephalosporin (3GC) β-lactam antibiotics. In brief, cultures were inoculated onto cetyltrimethylammonium bromide (CTAB)-amended nutrient agar plates at the recommended concentration of 50 mg L^−1^ and incubated for 48 h at 30 °C [20]. *E*. *coli* and *Pseudomonas spp*. with no known biocide resistance determinants were used as control strains. Strains simultaneously resistant to biocides and 3GCs were selected for further characterisation. 3GC resistance genes *bla*_CTX-M_, *bla*_*SHV*_ and *bla*_TEM_ and biocide resistance genes *qacH*, *qacE* and *qacEΔ1* were screened for as previously described [16, 20, 23]. Screened strains were then tested for the ability to transfer phenotypic biocide resistance to a naive *E*.* coli* recipient using *in vitro* conjugation assays as previously described [16]. In brief, modified *E*. *coli* DH10B (Str^R^Rif^R^) was used as a recipient strain for solid conjugal mating assays with biocide-resistant and cefotaxime-resistant strains used as donors. Transconjugants were selected using Luria Broth (LB) (Sigma Aldrich) plates amended with streptomycin (100 mg L^−1^), rifampicin (100 mg L^−1^) and CTAB (50 mg L^−1^). Donor strains and the recipient strain were plated separately onto streptomycin (100 mg L^−1^), rifampicin (100 mg L^−1^) and CTAB (50 mg L^−1^) LB selective plates as controls. Selected transconjugants were tested for biocide-resistant and 3GC-resistant phenotypes as previously described in addition to being screened for the 3GC genotype observed in the donors [16, 20, 23].

### Statistical analysis

All statistics were performed using Genstat 15th edition SP1 (VSN international). Proportions were compared using Fisher’s exact test. *χ*^2^ test was used for analysis of correlations between antibiotic resistance phenotypes. The Mann–Whitney *U* test was used for comparison of population numbers.

## Results

### Impact of WWTP effluent on CL1 abundance

**Fig. 1:**
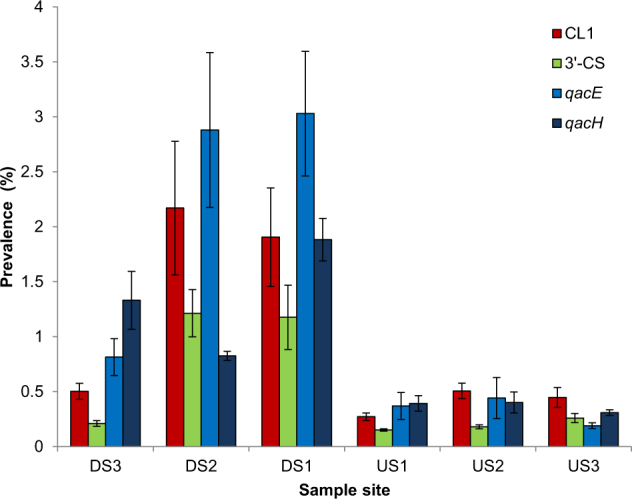
Prevalence of CL1s, 3’-CS region of CL1s, and QAC resistance genes *qacE* and *qacH*, as determined by qPCR for three sites downstream of WWTP Finham (DS1, DS2 and DS3) and three sites upstream of WWTP Finham (US1, US2 and US3). Error bars are two standard error units based on three biological replicates

A total of 18 samples were collected consisting of six sets of triplicates taken at 3 × 300 m intervals DS and US of Finham WWTP on the River Sowe [19]. There were no immediate inputs (>10 km) above the WWTP and previous chemistry analysis had indicated the US stretch of river to be low in chemical indicators of sewage [3]. To investigate the impact of WWTP effluent on CL1 prevalence in the total sediment communities, qPCR was performed on metagenomic DNA to quantify total gene prevalence for the CL1 integrase (*intI1*) (Fig. 1). WWTP effluent significantly increased CL1 abundance in river sediments DS (*χ*^2^ = 65,291, *P* < 0.0001), with the mean prevalence of combined DS sites (1.53%), a near fourfold increase on the CL1 prevalence at US sites (0.41%). There were variations in CL1 prevalence between DS sites depending on distance from the effluent source, but DS3 was still significantly higher than the mean US CL1 prevalence (*χ*^2^ = 994.993, *P* < 0.0001), indicating a continued impact of the WWTP on CL1 prevalence in the bacterial sediment communities ~1 km DS. To understand the relative abundances of different CL1 subsets, we quantified the *qacE∆1* 3′-CS motif to estimate the prevalence of 3′-CS CL1s  in comparison to lesser studied ‘atypical’ subsets (Fig. 1). As with the total CL1 community, 3′-CS CL1s had a significantly higher prevalence DS compared to US (*χ*^2^ = 42,333.536, *P* < 0.0001) (Fig. 1), however, a consistent 1:1 ratio of 3′-CS:atypical CL1s was reported across both DS and US sites. From this, we can determine that approximately 50% of CL1s belong to the 3′-CS subset across a range of polluted and unpolluted environmental sites, with the others being ‘atypical’.

### Comparative AMR profiling of river sediment communities

**Fig. 2:**
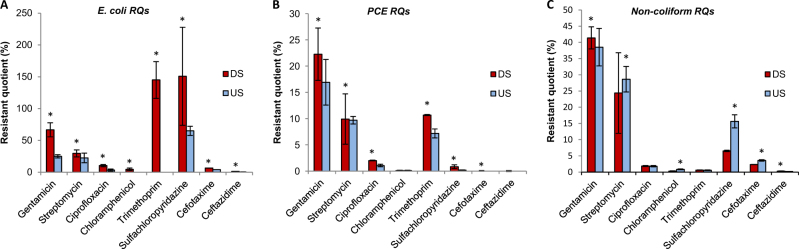
Resistant quotients for bacteria isolated from a river downstream of an effluent discharge in comparison to upstream. **a** *Escherichia coli*, **b** presumptive coliforms except for *E*. *coli*, **c** Gram-negative *Proteobacteria* (non-coliforms). * denotes significant differences

Changes in the antimicrobial resistant phenotypes of the bacterial sediment community were investigated by determining RQs across a range of antibiotics for three bacterial groups (Gram-negative non-coliforms, *E*. *coli* and PCEs (all other predicted coliforms excluding *E*. *coli*)) from DS and US sites (Fig. 2). A quarter of all bacteria isolated DS were resistant at clinically relevant breakpoints to streptomycin, gentamicin, trimethoprim and sulfachloropyridazine. Resistance profiles were heavily impacted by WWTP effluent, with RQs for the PCEs significantly higher at DS sites for all antibiotics except streptomycin, chloramphenicol and ceftazidime (*χ*^2^ (Supplementary Table 4) *P* < 0.0001), and presumptive *E*. *coli* RQs were significantly higher for all antibiotics at DS sites compared to US sites (*χ*^2^ (Supplementary Table 4) *P* < 0.0001). For two antibiotics (trimethoprim and sulfachloropyridazine), the selective effect for *E*. *coli* was so strong that it enriched the number of *E*. *coli* above what was previously recorded in the absence of antibiotic selection. In addition to increases in the percentage of antibiotic-resistant bacteria, DS sites had more than double the bacterial load of US sites, suggesting a large introduction of antibiotic resistant bacteria into the DS sites by the WWTP (Supplementary Table 5).

### Dissemination of CL1s in sediment communities

**Fig. 3:**
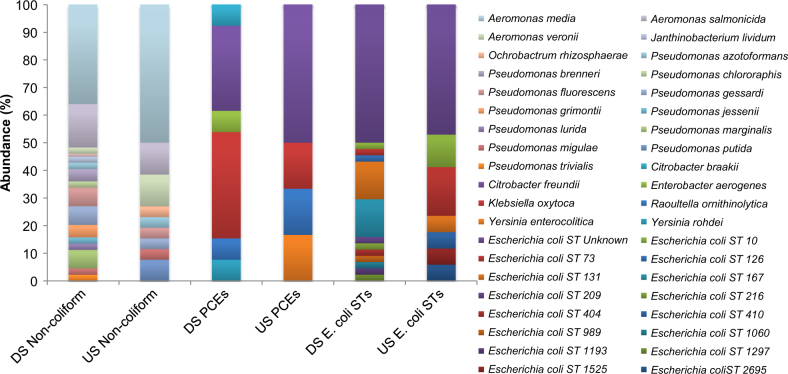
Species and sequence type composition of CL1-containing isolates broken into three bacterial groups: Gram-negative non-coliforms, presumptive coliforms excluding *E*. coli (PCEs) and *E*. *coli*. DS non-coliforms *n* = 44, US non-coliforms *n* = 26 DS PCEs *n* = 13, US PCEs *n* = 6, DS *E*. *coli* STs *n* = 45, US *E*. *coli* STs = 17

Although CL1s have been observed in non-clinical environments [2, 30], the extent to which they have spread throughout bacteria not commonly associated with the clinic remains poorly understood. With culture-independent analysis suggesting that DS sites had a large increase in the prevalence of CL1s, we undertook a large isolation effort from DS and US sites to compare impacted and non-impacted bacterial communities carrying CL1s. A total of 664 bacteria were isolated and screened for the presence of CL1s from pooled DS and US samples (348 DS and 316 US). CL1 carriage was significantly increased in DS antibiotic resistant isolates (43.28%) compared to US antibiotic resistant isolates (21.85%) (*χ*^2^ = 21.876, *P* < 0.0001), supporting culture-independent analyses that CL1 prevalence was significantly increased by WWTP effluent at DS sites. Across DS and US sites, CL1s were recovered from 26 species across four bacterial families showing the widespread dissemination of this genetic element (Fig. 3). Although more species were observed to carry CL1s DS (22) than US (15), this increase in diversity was not statistically significant. In addition to a range of commonly occurring river sediment bacteria (e.g. *Aeromonas spp*., *Janthinobacterium sp*. and *Ochrobactrum sp*.), CL1s were detected in a range of bacteria commonly associated with the clinic from both DS and US sites, including *E*. *coli*, *Klebsiella sp*., *Citrobacter sp*. and *Yersinia sp*.. MLST was used for further analysis of CL1-bearing *E*. *coli* populations; many STs were unique to each sediment site, with the pandemic strain ST131 being the most common DS. Surprisingly, this ST and two others (73,10) were observed both US and DS. A large proportion of both DS and US CL1 carrying *E*. *coli* had no known ST, suggesting they are not commonly associated with the clinic.

### Analysis of CL1 gene cassette diversity across bacterial isolates

**Fig. 4:**
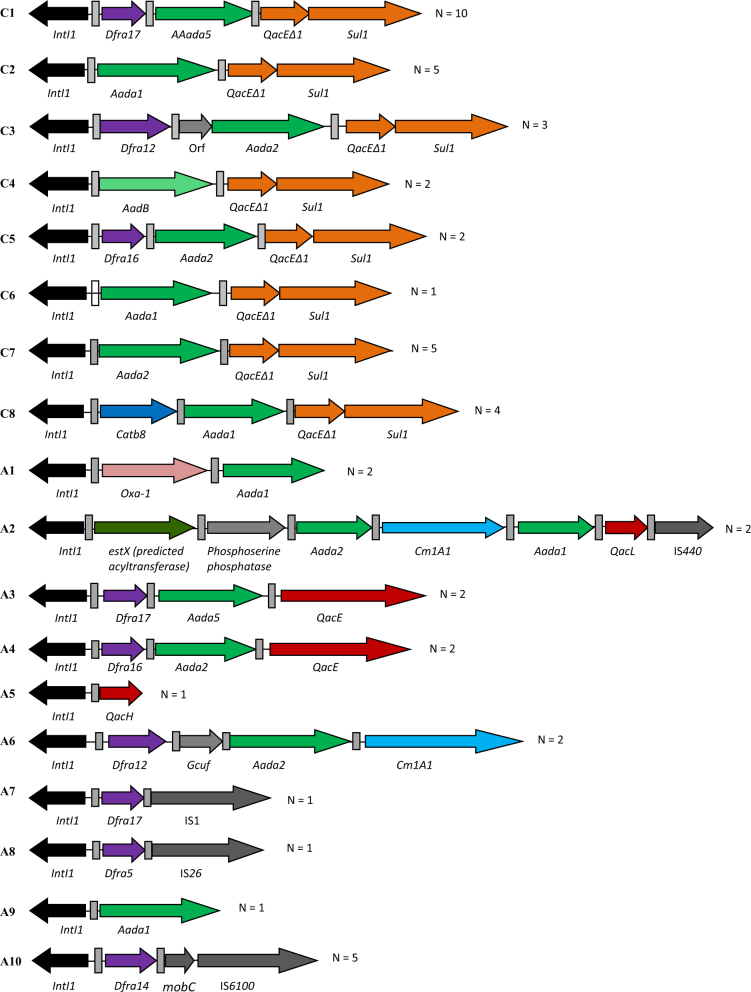
Schematic representation of the different types of gene cassette arrays found throughout CL1-bearing bacteria. CL1s with 100 % sequence nucleotide identity between identified features were grouped, with *N* = the number of isolates recovered carrying the specified gene cassette array. C1–C8 refer to clinical CL1s. A1–A10 refer to atypical CL1s. Recombination sites (attC and attI) are denoted by grey boxes. All sequences of CL1s have been submitted to Genbank (Accession MF686098-MF686115).

To investigate the differences in CL1 gene cassette arrays DS compared to US, and to further our understanding of the link between CL1s in common environmental isolates compared to more clinically associated isolates, we characterised our CL1-containing isolates using a novel two-step PCR method (Fig. 4). 57 % of 3′-CS CLs were successfully amplified and sequenced with eight unique arrays identified, two of which were novel to this study (C6 and C8) (Fig. 4, Table 1). In addition to 3′-CS CL1s, 10 unique atypical CL1 gene cassette arrays from 20 isolates were characterised, with three being novel to this study (A5, A7, A8) (Fig. 4, Table 1). Sixteen different gene cassettes were found associated with the ten atypical CL1s, giving rise to ten potential phenotypes including QAC resistance. This is more than double the number of predicted phenotypes observed from 3′-CS CL1s (4) despite being from a smaller sample size. Throughout both atypical and 3′-CS CL1 gene cassette arrays, genetic linkage of multiple resistance genes was observed, with as many as three antibiotic resistance genes carried in one gene cassette array (Fig. 4), and QAC resistance genes regularly co-carried with antibiotic resistance genes.

**Table 1:** Summary of characterised CL1 gene casette arrays

	DS species, observed number	US species, number observed
3′-CS CL1s		
C1	*E*. *coli* ST1193, *E*. *coli* ST209, *E*. *coli* ST404, *E*. *coli* ST unknown *(2)*, *K*. *oxytoca*	*E*. *coli* ST73, *E*. *coli* ST28, *A*. *media (2)*
C2	*A*. *media (2)*, *A*. *salmonicida*, *E*. *coli* ST (unknown)	*E*. *coli* ST2695
C3	*R*. *ornithinolytica*, *C*. *freundii*	*C*. *freundii*
C4	*C*. *freundii*	*R*. *ornithinolytica*
C5	*E*. *coli* ST1193, *E*. *coli* ST unknown	None observed
C6	*A*. *media*	None observed
C7	None observed	*A*. *salmonicida (2)*, *A*. *media*, *P*. *putida*, *C*. *freundii*
C8	*E*. *coli* ST unknown, *A*. *media (2)*, *A*. *salmonicida*	None observed
*Atypical CL1s*		
A1	*E*. *coli* ST10	*E*. *coli* ST73
A2	*E*. *coli* ST unknown, *E*. *coli* ST1060	None observed
A3	None observed	*E*. *coli* ST unknown (2)
A4	None observed	*E*. *coli* ST unknown (2)
A5	*P*. *gessardi*	None observed
A6	*E*. *coli* ST989	*A*. *media*
A7	*E*. *coli* ST131	None observed
A8	None observed	*A*. *sobria*
A9	None observed	*A*. *media*
A10	*E*. *coli* ST131 (2), *E*. *coli* ST unknown, *C*. *freundii*	*C*. *freundii*

Table of CL1 gene cassette arrays characterised and the bacteria associated with each one. Bacteria were isolated from aquatic sediments (DS) and upstream (US) of a WWTP outflow. ST unknown—does not match to anything in database

Further analysis of the distribution of unique sequenced CL1s revealed half of the unique CL1s were shared between different species and sites (Table 1), with commonly reported river sediment bacteria frequently carrying identical CL1s to those observed in clinically associated bacteria (Fig. 1, Table 1, Supplementary Table 1). In particular this is exemplified in the atypical CL1s by A10 which was recovered from *Aeromonas media* and *Citrobacter freundii* at US sites, and the pandemic *E*. *coli* clone ST131 at DS sites. The sharing of CL1s between environmental and human-associated bacteria was also prevalent in the 3′-CS CL1s, exemplified by C1 which was recovered at US sites from *A*. *media* and two clinically associated *E*. *coli* STs (ST1193, ST404), and recovered at DS sites from two clinically associated *E*. *coli* STs (ST73 and ST28) and *K*. *oxytoca*.

Supporting our observations from the culture-independent analyses, 40 % of CL1-bearing isolates contained the 3′-CS motif suggesting at least half of the CL1s at the tested sites lack the 3′-CS. In addition, the diversity of hosts recovered containing atypical or 3′-CS CL1s did not differ significantly between CL1 types with both 3′-CS and atypical CL1s frequently recovered from human associated and commonly reported environmental bacteria (Table 1, Supplementary Table 1). Such results demonstrate that both common clinically and environmentally associated bacteria can carry either 3′-CS or atypical CL1s in the riverine environment, with no CL1 group biased to a particular bacterial population.

### Determining the impact of WWTP effluent and CL1 type on MIC profiles

**Fig. 5:**
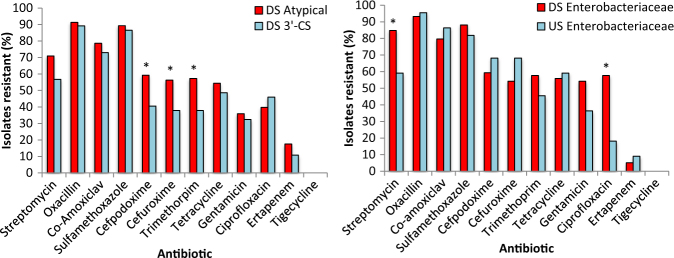
Comparison of the number of isolates resistant to a panel of 12 tested antibiotics for (**a**) DS Atypical CL1s, vs. DS 3′-CS CL1s (**b**) DS *Enterobacteriaceae* vs. US *Enterobacteriaceae*. * denotes significant differences

To determine whether CL1-bearing bacterial isolates DS were resistant to more antibiotics than CL1-bearing bacterial isolates US, MIC susceptibility profiling of individual CL1-containing isolates was performed to 12 antibiotics (Fig. 5; Supplementary Table 1). In particular, the Enterobacteriaceae showed significant increases in resistance to ciprofloxacin (*χ*^2^ = 10.08, *P* = 0.0015) and streptomycin (*χ*^2^ = 6.06, *P* = 0.014) DS compared to US (Fig. 5). On average isolates were individually resistant to over 50 % of tested antibiotics from both DS and US sites (6.45 DS, 6.05 US), demonstrating how CL1-bearing isolates exhibit multi-resistant phenotypes. Concerningly, some isolates were simultaneously resistant to as many as 10 of the 12 antibiotics tested. A comparison was made between atypical CL1 profiles and 3′-CS CL1 profiles to investigate if there were any phenotypic differences between isolates which carried different CL1 subsets (Fig. 5). Surprisingly despite the 3′-CS CL1s commonly referred to as being ‘clinical’ CL1s [31], DS atypical CL1-bearing isolates had a higher prevalence of resistance to more antibiotics than their 3′-CS counterparts. For example, atypical CL bearing isolates had a significantly higher prevalence of cefpodoxime (*χ*^2^ = 4.684, *P* = 0.0304) and cefuroxime resistance (*χ*^2^ = 5.54, *P* = 0.0196) compared to 3′-CS CLs. Differences in AMR phenotypes were compared between bacterial groups to determine if the human-associated Enterobacteriaceae had broader AMR profiles than the common river sediment Proteobacteria (e.g. *Aeromonas* spp., *Ochrobactrum* sp., *Janthinobacterium* sp., *Pseudomonas* spp.). No significant differences were observed in the total prevalence of AMR phenotypes between Aeromonodaceae, Pseudomonadaceae or Enterobacteriaceae indicating the CL1-containing environmental isolates were as resistant as their clinical counterparts. This may be in part due to intrinsic resistance mechanisms commonly associated with non-coliforms. Pattern-based analysis of resistance revealed DS resistant phenotypes showed significant linkage with the creation of clusters of resistance (Supplementary Tables 2 and 3), such as ertapenem which significantly correlated with six other resistances including ciprofloxacin and gentamicin (Supplementary Tables 2 and 3). US correlations were evident, though these were different to those observed DS of the WWTP and often consisted of smaller groups of resistance phenotypes clustering together.

### The role of quaternary ammonium compounds in the selection of environmental AMR

Analysis of chemistry data previously collected at the site showed a significantly higher abundance of the detergent marker boron DS compared to US (Mann–Whitney *U*, *P* < 0.05) [3, 32], supporting previous surveillance studies reporting mg/L quantities of biocidal detergents in waste water [33–35]. Prior studies have suggested that biocides such as QACs may select for CL1s based on selection for the QAC resistance genes *qacE∆1*, *qacE* and *qacH* [20, 31]. Initially using qPCR methods for enumerating common CL1 associated QAC resistance genes [23], we tested the hypothesis that biocides from the effluent are contributing to elevated CL1 levels in the DS total community (Fig. 1). QAC resistance genes were significantly more abundant DS compared to US with the mean *qacE* prevalence of combined DS sites almost tenfold higher than the mean of the US combined sites (*χ*^2^ = 143,796, *P* < 0.0001), and the mean *qacH* prevalence of all sites fivefold higher DS compared to US (*χ*^2^ = 5636, *P* < 0.0001). The impact of effluent on the abundance of QAC resistance genes was sustained over 1 km DS of the WWTP with the prevalence of both *qacE* and *qacH* not significantly decreasing with distance from the WWTP. We next determined whether increased prevalence of QAC resistance genes observed in the total community were linked to AMR genes in our isolates. Four unique QAC resistance genes were contained in the sequenced CL1 gene cassette arrays (*qacE*, *qacE∆1*, *qacH* or *qacI*), with 11 of the 18 unique CL1 gene cassette arrays sequenced carrying a QAC resistance gene alongside a gene cassette conferring an antibiotic resistance phenotype. Considering the abundance of each sequenced CL1 subset (Table 1), this translates to 75 % of CL1-containing isolates co-carrying a biocide resistance gene alongside an AMR resistance gene. Furthermore phenotypic QAC resistance testing of CL1 isolates suggested 89 % were resistant to biocides and grew unimpaired to 50 mg L^−1^.

Finally, to test whether a QAC could solely select for the transfer of an antibiotic resistance gene and phenotype to a sensitive strain, we selected 48 QAC resistant isolates for conjugal transfer experiments, which were also resistant to multiple antibiotics including 3rd generation cephalosporin (3GC) β-lactams. Isolates with 3GC resistance were preferentially chosen on the basis that 3GC resistance has been widely reported across different aquatic environments and is an antibiotic of high clinical importance [14, 16, 36]. Using the QAC resistant strains as donors we demonstrated 42 % could successfully transfer their QAC resistant phenotype to a naive recipient *E*. *coli*, with 75 % (15/20) of the harvested transconjugants also resistant to clinical levels of the 3GC antibiotics cefotaxime and ceftazidime [24]. Donors were screened for the mechanism of 3GC resistance, which revealed they all carried *bla*_CTX-M_. Transconjugants were confirmed to be positive for the presence of this gene, in addition to being positive for CL1s. Thus, in summary, for 15/48 β-lactam and QAC resistant strains, we successfully demonstrated that QACs select for the mobilisation of both CL1s and *bla*_CTX-M_.

## Discussion

In the current global AMR crisis it is essential to understand the link between the environment and the clinic, and to identify key selecting factors for AMR gene dissemination. Here we demonstrate the widespread impact which WWTP effluent has on increasing both phenotypic and genotypic levels of AMR in the entire sediment microbial community, supporting the hypothesis that WWTP effluent is a major driver of AMR in aquatic environments [3, 16]. Previous studies have suggested the environmental and human resistomes are shared [22], we believe this study to be the first to isolate identical complex genetic resistance determinants from both human associated and common environmental bacteria across connected sites. Such findings are clear evidence that the same AMR genes and associated genetic elements circulate between common environmental bacteria and human-associated bacteria, supporting the hypothesis that AMR in the environment is inextricably linked to AMR in the clinic [37].

CL1s are clinically important genetic elements with the ability to confer resistance to multiple antibiotics [5]; however, a detailed environmental study of their gene cassette diversity to date has been hampered due to biases against amplifying variable regions without the 3′-CS [9, 10]. We successfully pioneered a new technique for studying CL1s with the analysis of CL1 ecology revealing approximately only half of CL1s in river sediments contain the classic 3′-CS architecture [4]. This is the first estimate of the atypical CL1 population in the wider environment, and supports the argument that the diversity of CL1s and their associated gene cassettes has to date been underestimated [9–11], perhaps in part due to a reliance on primer sets targeting the 3′-CS [7, 8]. In addition to providing genetic novelty, atypical CL1s were invariably demonstrated to contribute to isolates AMR profiles as much as their 3′-CS counterparts.

AMR genes and phenotypes in the riverine environment are clearly increased in prevalence and abundance by WWTP effluent as demonstrated in this study and others prior [3, 16, 19]; having established that this anthropogenic input drives levels of AMR in riverine systems, we demonstrated a potential key selection pressure in effluent is QAC detergents. There are numerous potential mechanisms by which AMR genes could persist in the environment, for example sub MIC concentrations of antibiotics can select for plasmid maintenance [38], and plasmids carrying AMR genes and CL1s may yield no detectable fitness cost [39]. Here, our findings that QACs detergents could co-select for the transfer of mobilised antibiotic resistance genes, supports the hypothesis that pollutants other than antibiotic residues can select for antibiotic resistance [20, 23, 31]. Recent genomic surveys have proven antibiotic resistance genes can co-occur frequently with metal resistance genes [21, 40]. No metal resistance genes were recorded in sequence data here, however QAC resistance genes were observed to frequently co-occur with antibiotic resistance genes, which is likely a reflection of QACs being a greater selective pressure for adaptive genetic elements at sewage polluted sites than metals. This hypothesis is supported by previous observations of QAC resistance genes in detergent rich and sewage polluted environments [20, 23], observations of high levels of QACs in sewage [33, 35], previous reports of high levels of boron (a common detergent marker) at DS sample sites in this study [3, 15], and our culture-independent data suggesting a significant increase in prevalence of QAC resistance genes in the whole riverine sediment community 1 km DS of the WWTP.

There are two possible mechanisms by which QAC resistance gene prevalence could be increased DS of the WWTP. Selection for QAC resistance genes could be occurring in the WWTP, with QAC resistant bacteria and their associated resistance genes being introduced into the river in the effluent. The introduced resistance genes could then be selected by QACs in the effluent in the river *in situ*. Alternatively, QACs in the effluent could be selecting for QAC resistance genes already present within the river. In both of these proposed scenarios, mobilised QAC resistance genes may be selected for by QACs in the effluent. In addition to QACs, other factors such as nutrients in the effluent could also be impacting the microbial ecology of the river [41].

The main aim of this study was to use CL1s as a marker genetic element to investigate the extent of AMR gene dissemination in riverine bacterial communities, and to understand how and why environmental reservoirs of AMR form. In conclusion, WWTP effluent was responsible for large changes in the river sediment bacterial community’s AMR phenotypes and genotypes. The introduction of human-associated bacteria by the WWTP effluent played a key part in changing the resistome of the river; however, the scale to which CL1s were distributed and shared across human associated and environmental bacteria is also suggestive of bacteria from different origins sharing mobile elements. Our study highlighted a key role which QACs may play in selecting for environmental antibiotic resistance, establishing a clear mechanism for antibiotic resistance gene selection and persistence in the environment in the absence of antibiotic selection. Ultimately increased selection for AMR in the wider environment is a cause for clinical concern, as it can drive the emergence of novel resistance genes in clinically associated bacteria as well as providing a potential health risk though direct exposure [14, 37].

## Electronic supplementary material

Supplementary Information

Supplementary Table 1

Supplementary Table 2

Supplementary Table 3

Supplementary Table 4

Supplementary Table 5
