# Supplementary material for: The widespread dissemination of integrons throughout bacterial communities in a riverine system
Source: ISME J. 2018 Jan 26;12(3):681–91. doi: 10.1038/s41396-017-0030-8 (PMC5864220; doi:10.1038/s41396-017-0030-8)
Supplement: Supplementary file 1 — Supplementary Information [file 41396_2017_30_MOESM1_ESM.docx]

**Supplementary Information**

**Methodology for characterization of CL1s**

3’-CS CL1s were detected and characterised using published primers HS915 5’ - GTGCCGTGATCGAAATCCAG-3’ and HS550 5‘- 30 CTAGGCATGATCTAACCCTCGG- 3’ (Marquez *et al.,* 2008). Conditions were optimised for long range PCR. Reactions consisted of a 50 µl reaction using Takara Ex Taq (Clontech) reagents including 0.25µl taq, 5 µl reaction buffer, 5 µl dNTPs, 2 µl DNA template, and primers at a concentration of 0.8 µΜ. PCR cycle conditions: stage 1, 95ºC 2 mins; stage 2, 35 cycles of 95ºC 30 s, 60ºC 30 s, 72ºC 8 mins; stage 3 72ºC for 10 mins. Subsequent PCR products were analysed analysed using a 1 % agarose (BioRad) gel and purified (Qiagen) for sanger sequencing (Macrogen, Korea).

Analysis of atypical CL1s was performed using a long range two-step gene walking method^28^, modified to be CL1 specific. Reactions consisted of a 50 µl reaction using Takara Ex Taq (Clontech) reagents including 0.25µl taq, 5 µl reaction buffer, 5 µl dNTPs, 2 µl DNA template, and primer HS915 at a concentration of 1.5µΜ primer. PCR cycle conditions were: stage 1 94 ºC 4 mins; stage 2, 30 cycles of 94 ºC 30 secs, 60 ºC 30 secs, 72 ºC for 6 mins; stage 3, 94 ºC 30 secs, 40 ºC 30 secs, 72 ºC for 6 mins; stage 4, 30 cycles of 94 ºC 30 secs, 60 ºC 30 secs; stage 5, 72 ºC 10 mins. PCR products were separated using gel electrophoresis on a 1 % agarose gel (Biorad) and distinct bands were purified (Qiagen). Sanger Sequencing was performed (Macrogen, Korea) using a newly designed nested primer IntSeq 5’- GAGGATGCGACCACTTCAT - 3’.

**Methodology for total community CL1 and *qac* gene quantification**

CL1s and associated genes were quantified as previously described by *Gaze et al.* 2011. The quantification of CL1s was performed using primer pair Int1f2 *5’-*TCGTGCGTCGCCATCACA-3’ and Int1R2 5’-GCTTGTTCTACGGCACGTTTGA-3’. For quantification of *qacE* primer pair QacEfor 5’-CTTCATGGGCAAAAGCTTGATG-3’ QacErev 5’-TTAGTGGGCACTTGCTTTGGA-3’was used. For quantification of *qacE∆1* primer pair QacEcom1f 5’-GTTATGGCATCGCATTTTATTTTCT-3’ and QacEcom1r 5’-CCGACCAGACTGCATAAGCA-3’ was used with a subsequent subtraction of the number of amplicons from the number of *qacE* amplicons to yield a final value*. QacH* was quantified using primer pair *QacHf2 5’-* TGGCAGCTATTGCTTGGATTT – 3’ and *QacHr2 5’TGCCAATGAACGCCCAGAAG -3’.* For quantification of 16S rRNA primers 1369f 5’-CGGTGAATACGTTCYCGG-3’ and 1492R 5’-GGWTACCTTGTTACGACTT-3’ were used. All reactions were performed using Power SYBR Green PCR Mastermix (Applied Biosystems) as per manufactures instructions and as published (Gaze *et al.* 2011). Standards were created for absolute quantification as published (Gaze *et al.* 2011).
