# Supplementary material for: The widespread dissemination of integrons throughout bacterial communities in a riverine system
Source: ISME J. 2018 Jan 26;12(3):681–91. doi: 10.1038/s41396-017-0030-8 (PMC5864220; doi:10.1038/s41396-017-0030-8)
Supplement: Supplementary file 3 — Supplementary Table 2 [file 41396_2017_30_MOESM3_ESM.docx]

Supplementary Table 2: Correlations between different antibiotic resistant phenotypes in DS isolates

| **Antibiotic resistant phenotype** | **Correlations with other antibiotic resistant phenotypes** |
| --- | --- |
| Streptomycin | Trimethoprim, gentamicin, tetracycline, sulfamethoxazole |
| Ciprofloxacin | Ertapenem, gentamicin, co-amoxyclav, oxacillin |
| Cefpodoxime | Ertapenem, cefpodoxime, cefuroxime, co-amoxyclav, oxacillin |
| Cefuroxime | Trimethoprim, ertapenem, cefpodoxime, co-amoxyclav, oxacillin |
| Trimethoprim | Cefuroxime, ertapenem |
| Ertapenem | Ciprofloxacin, cefpodoxime, cefuroxime, trimethoprim, gentamicin, co-amoxyclav   |
| Tetracycline | Streptomycin, sulfamethoxazole, oxacillin |
| Sulfamethoxazole | Streptomycin, tetracycline |
| Co–amoxyclav | Ciprofloxacin, cefpodoxime, cefuroxime, ertapenem, oxacillin |
| Oxacillin | Cefpodoxime, cefuroxime, gentamicin, tetracycline, co-amoyclav |
