# Supplementary material for: The widespread dissemination of integrons throughout bacterial communities in a riverine system
Source: ISME J. 2018 Jan 26;12(3):681–91. doi: 10.1038/s41396-017-0030-8 (PMC5864220; doi:10.1038/s41396-017-0030-8)
Supplement: Supplementary file 4 — Supplementary Table 3 [file 41396_2017_30_MOESM4_ESM.docx]

Supplementary Table 3: Correlations between different antibiotic resistant phenotypes in US isolates

| **Antibiotic resistant phenotype** | **Correlations with other antibiotic resistant phenotypes** |
| --- | --- |
| Streptomycin | Cefuroxime |
| Ciprofloxacin | None |
| Cefpodoxime | Cefuroxime, trimethoprim |
| Cefuroxime | Cefpodoxime, trimethoprim, gentamicin |
| Trimethoprim | Cefpodoxime, cefuroxime, ertapenem |
| Ertapenem | Trimethoprim |
| Gentamicin | Cefpodoxime, tetracycline |
| Tetracycline | Gentamicin, Sulfamethoxazole |
| Sulfamethoxazole | Tetracycline |
| Co–amoxyclav | Oxacillin |
| Oxacillin | Co-amoxyclav |
